# QoS in Wireless Sensor Networks

**DOI:** 10.3390/s18113983

**Published:** 2018-11-16

**Authors:** Nathalie Mitton

**Affiliations:** Inria, 40, avenue Halley, 59650 Villeneuve d’Ascq, France; nathalie.mitton@inria.fr

The last decades have witnessed advances in multiple wireless sensor networks in both the academic and industrial world. A wireless sensor network (WSN) is composed of a set of distributed hardware-constrained wireless devices in charge of monitoring targeted areas. The applications of WSNs are numerous and range from environmental monitoring to urban health monitoring through healthcare, logistic applications, and smart grids, to name a few. The growing usage of wireless sensors in different scenarios makes the quality-of-service (QoS) a paramount issue in wireless sensor applications.

Each application features its own requirements in terms of QoS. And in each application, information may request different QoS processing (regular monitoring messages vs. alarm messages). However, due to the unreliable characteristics of the wireless medium and the hardware limitations of devices, providing QoS in WSN-based applications remain a challenging task.

This Special Issue called for high quality, up-to-date, innovative, original, current advances in QoS management in wireless sensor networks in general and in wireless technologies for Internet of Things (IoT).

## Contributions

This Special Issue attracted a large number of high-quality contributions. The reviewing process selected the ten best submissions that address several complementary facets of QoS in wireless sensor networks for different applications. These submissions have been proposed by authors from France, Korea, Russia, Qatar, Kuwait, China, Belgium, Japan, and the USA. The QoS criteria that they generally aim to improve include delay, energy consumption, resiliency, reachability, and packet losses. In the following, a summary of the scope and main contributions of each of these papers is provided as a teaser for the interested reader.

Reference [1,2,3] focus on wireless sensor networks in general and aim to improve delay, energy, and/or link quality. These references propose novel cross-layer (MAC/NET) protocols to reduce the routing complexity and reachability in the network by taking account of the duty cycle. Ref. [1] proposes an opportunistic approach, ODYSSEY, and assesses its performance through extensive experimentation. Ref. [2] introduces data aggregation in the routing protocol to reduce the data to be sent (and, thus, interference and energy) and selects the next hop based on the data queue status.

Two contributions [4,5] focus on the use of wireless sensor networks for operating smart grids. Smart grids must handle simultaneously different traffic with different QoS requirements over the same network. Ref. [4] proposes a novel metric to be used by the smart grid standard routing protocol RPL to allow traffic differentiation and demonstrates its performance in terms of delay through real experimentation. Ref. [5] discusses the use of multiple technologies for the WSN in the smart grid, highlighting the strengths and limitations.

Reference [6] considers a wireless sensor-actor network and proposes a novel self-healing mechanism that ensures a minimum resiliency and the reliability of the network. This approach leverages the ability of some nodes to move to re-build a network topology with minimum cost when a failure is detected.

Reference [7] focuses on Wi-Fi HaLow, an adaptation of the widespread Wi-Fi technology for the Internet of Things scenario, and, in particular, aims to reduce the contention time between stations when connecting.

Other papers consider less traditional wireless sensor networks that are either based on cognitive radio [8], wireless rechargeable sensors [9], or wake-up radio sensors [10]. These approaches address very new technologies and leverage new hardware features to optimize some QoS criteria.

Altogether, these outstanding contributions constitute this MDPI Sensors Special Issue. While this special issue is far from delivering a complete coverage of this exciting research area, we hope that these samples give the audience a taste of the main trends and research directions in this area, and provide the audience with an opportunity to explore and collaborate in related fields.
